# Basal Cell Carcinoma of the Cloacal Aperture in a Red Kangaroo (*Osphranter rufus*)

**DOI:** 10.3390/vetsci12121145

**Published:** 2025-12-01

**Authors:** Louise van der Weyden, Agustina Fitte, Nicolize O’Dell

**Affiliations:** 1 Wellcome Sanger Institute, Wellcome Genome Campus, Hinxton, Cambridge CB10 1SA, UK; lvdw@sanger.ac.uk; 2 Centre for Wildlife Health, Pretoria National Zoological Gardens, South Africa Biodiversity Institute, Pretoria 0001, South Africa; a.fitte@sanbi.org.za; 3 Department of Paraclinical Sciences, Faculty of Veterinary Science, University of Pretoria, Onderstepoort 0110, South Africa; 4 Centre for Veterinary Wildlife Studies, Faculty of Veterinary Science, University of Pretoria, Onderstepoort 0110, South Africa

**Keywords:** kangaroo, macropod, tumour, basal cell carcinoma, skin, cloaca

## Abstract

There have been few reports of neoplasia in kangaroos to date. In this report, we describe a 13-year-old male captive Red kangaroo (*Osphranter rufus*) that developed a mass on the right side of the cloacal aperture. Histopathological analysis of a biopsy of the mass revealed proliferating, neoplastic epithelial islands with necrotic centres infiltrating a moderately desmoplastic dermis. The islands were generally large with extensive central caseation necrosis consisting of cellular debris and innumerable neutrophils. The neoplastic epithelial cells resembled the basal cells observed in the basal layer of the epidermis and were characterised by scant to moderate eosinophilic cytoplasm with indistinct cytoplasmic margins. The diagnosis was basal cell carcinoma (BCC). This is the first report of a BCC in a kangaroo, and we compare the findings with that seen in dogs, cats and humans.

## Simple Summary

There have been few reports of neoplasia in kangaroos to date. In this report, we describe an adult male captive Red kangaroo (*Osphranter rufus*) that developed a mass on the opening of the cloaca, near the base of the tail. Histopathological analysis of the mass revealed large islands of neoplastic epithelial cells infiltrating into the dermis, which itself was showing signs of desmoplastic change. The central area of the islands was filled with cellular debris and numerous neutrophils. The neoplastic epithelial cells resembled the basal cells found in the basal layer of the epidermis and showed a scant-to-moderate cytoplasm with indistinct cytoplasmic margins. The diagnosis was basal cell carcinoma (BCC). This is the first report of a BCC in a kangaroo, and we compare the findings with BCC seen in dogs, cats and humans.

## 1. Introduction

Basal cell carcinoma (BCC) is a low-grade malignant neoplasm arising from the basal cells of either the interfollicular epidermis or the hair follicles, and is characterised by a lack of epidermal or adnexal differentiation [1]. In both humans and animals, BCC is locally invasive, typically showing extensive infiltration of the dermis and subcutaneous tissue often accompanied with epidermal ulceration. Metastasis is very rare in these cases, although recurrence at the surgical site can occur [1].

To date, there are only a handful of reports of neoplasia in kangaroos, including both the Eastern Grey kangaroo (*Macropus giganteus*) and the Red kangaroo (*Osphranter rufus*). These consist of a retrospective survey of Kansas City Zoo records between 1992 and 2002 [2], and five individual case reports [3,4,5,6,7]. Taken together, they have reported benign tumours, including trichoepithelioma [4,5] and lipoma [2,5]. Malignant tumours included oral squamous cell carcinoma [2,3], mammary carcinoma [2] and T-cell lymphoma [2]; while metastatic tumours included angioleiomyosarcoma with lung metastasis [7] and a metastatic adenocarcinoma of unknown primary origin [6]. A more recent retrospective study characterising spontaneous mammary gland tumours in over 1,000 macropod cases submitted to Northwest ZooPath between 1996 and 2019 found that Red kangaroos were most commonly diagnosed with mammary tubular carcinoma, either of the luminal-A or normal-like molecular subtype, with 2/11 of the cases showing pulmonary metastases [8]. In this report, we present the details of the case of basal cell carcinoma and compare the findings with that seen in canine, feline and human BCCs.

## 2. Case Report

**Figure 1:**
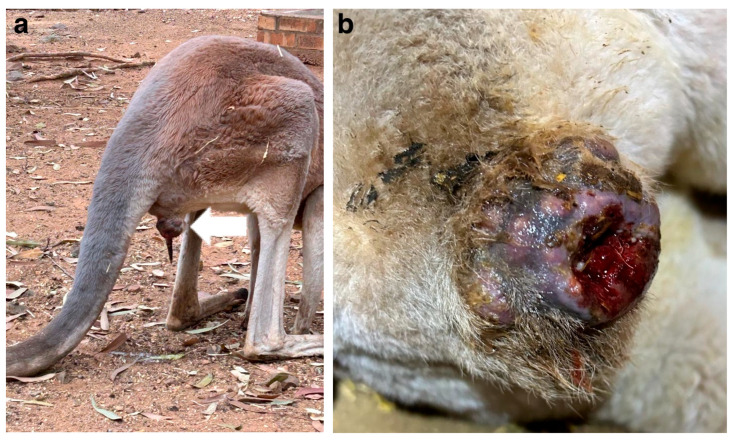
Macroscopic image of the mass on the cloacal aperture of the Red kangaroo. (**a**) Lateral-to-rear view of the kangaroo in the enclosure, with the arrow indicating the mass. (**b**) Close-up lateral-to-rear image of the mass.

A 13-year-old intact male Red kangaroo (*Osphranter rufus*) from the National Zoological Garden in Pretoria (South Africa) developed a large mass on the right side of the cloacal aperture (Figure 1a,b). The kangaroo was anaesthetised using 120 mg Ketamine (Kyron Prescriptions, Benrose, South Africa), combined with 2 mg Medetomidine (Kyron Prescriptions) and 10 mg Midazolam (Dazonil; Wildlife Pharmaceuticals, White River, South Africa). An incisional biopsy (1 cm^3^) of the mass was obtained and submitted to the Histopathology Laboratory of the Faculty of Veterinary Science, University of Pretoria for routine histopathology. The mass was then sprayed with Biotaine (5% Chlorhexidine Gluconate Concentrate Solution; Braun Medical, Randburg, South Africa) and 8mg of F10 Germicidal Barrier Ointment (Quaternary ammonium compounds 0.44% and cypermethrin 0.25%; Health and Hygiene, Florida Hills, 1716, South Africa) was applied, for disinfectant and insect repellent purposes, and the kangaroo released back into the enclosure.

**Figure 2:**
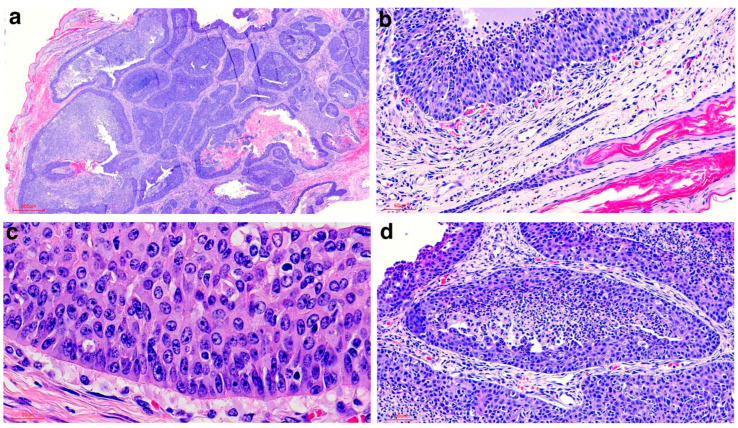
Histologic findings of the biopsy from the cloacal aperture mass of the Red kangaroo. (**a**) Neoplastic epithelial islands with necrotic centres infiltrating a moderately desmoplastic dermis and extending to the surgical margins in all areas (HE stain, 20× magnification). (**b**) Neoplastic cells resembling the basal cells observed in the basal layer of the adjacent hair follicles (HE stain, 200× magnification). (**c**) Round-to-oval, finely-stippled, basophilic nuclei containing mostly one single prominent central amphophilic nucleolus. Palisading is also observed (HE stain, 600× magnification). (**d**) Large islands with extensive central caseation necrosis (HE stain, 200× magnification).

The biopsy samples were fixed in 10% neutral buffered formalin, embedded in paraffin wax and routinely sectioned for histopathological evaluation. Examination of all the haematoxylin and eosin (HE)-stained sections (4 μm) revealed numerous, variably-sized, densely-cellular, unencapsulated, proliferating, neoplastic epithelial islands with necrotic centres infiltrating a moderately desmoplastic dermis and extended to the surgical margins in all areas (Figure 2a). The neoplastic epithelial cells resembled the basal cells observed in the basal layer of the epidermis and hair follicles and were characterised by scant-to-moderate eosinophilic cytoplasm with indistinct cytoplasmic margins (Figure 2b). The nuclei were round to oval, finely-stippled, basophilic and contained mostly one single prominent central amphophilic nucleolus (Figure 2c). The islands were generally large with extensive central necrosis consisting of cellular debris and innumerable neutrophils (Figure 2d), some being present between the neoplastic cells in the areas where the cells were still viable, giving the neoplastic cell layer a papillary appearance in certain areas. Palisading was occasionally evident on the periphery of the neoplastic islands. The mitotic rate averaged 60 mitoses in 2.37 mm^2^ (10 contiguous fields of view under 40× magnification). Small foci of squamous differentiation were noted rarely. Pigmentation and keratinisation were not a feature in this neoplasm. No direct communication with the overlying epidermis was evident in the examined sections; however, in certain areas, the basal layer of the epidermis appeared slightly dysplastic with occasional rete peg formation. The diagnosis was basal cell carcinoma (BCC).

**Figure 3:**
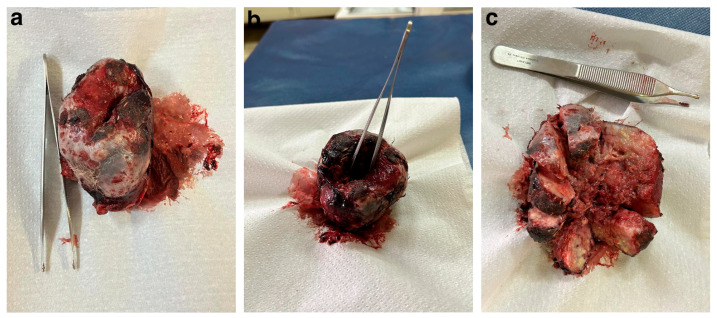
Macroscopic image of the mass surgically removed from the cloacal aperture of the Red kangaroo. (**a**) Overhead view of the tumour. (**b**) Overhead view of the tumour with tweezers demonstrating the collapsed central part of the tumour. (**c**) Dissection of the tumour to demonstrate the necrotic centre. The tweezers in the photos are 12cm in length.

Three weeks later, the kangaroo was then anaesthetised (as described above) and the mass surgically removed. Macroscopic examination of the mass showed it to be a brown, bloody and ulcerating doughnut-shaped lesion (approximately 6 × 5 × 4 cm in size; Figure 3a), with a central collapsing area of necrotic tissue (Figure 3b,c).

**Figure 4:**
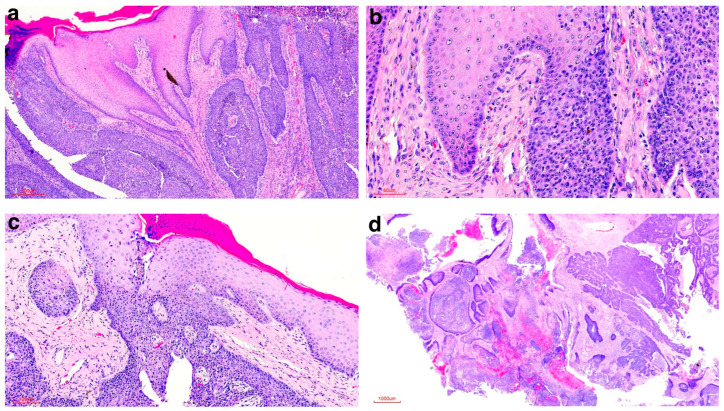
Histologic findings of the surgically removed tumour from the cloacal aperture of the Red kangaroo. (**a**) Areas of direct communication with the overlying epidermis at low magnification (HE stain, 40× magnification). (**b**) Area of direct communication with the overlying epidermis at higher magnification (HE stain, 200× magnification). (**c**) Area of direct communication with the overlying epidermis at higher magnification (HE stain, 100× magnification). (**d**) Neoplastic islands extended to all the surgical margins (HE stain, 10× magnification).

Histopathological analysis of the mass revealed the same findings as were seen in the original biopsy; however, numerous areas of direct communication with the overlying epidermis were evident in multiple areas (Figure 4a–c). Unfortunately, the neoplastic islands extended to all the surgical margins (Figure 4d).

**Figure 5:**
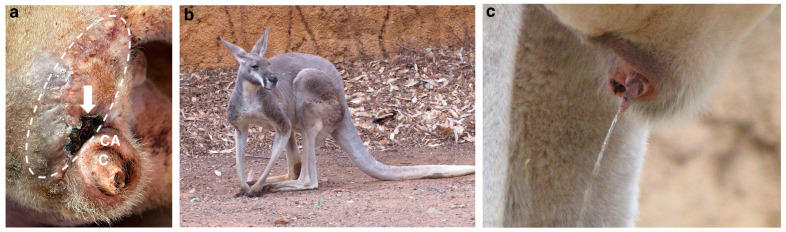
Macroscopic image of the surgical wound of the Red kangaroo at different times post-surgery. (**a**) Six-weeks post-surgery. The white line demarcating where the tumour was located before excision. The arrow points to a slight dehiscence of sutures followed by scab formation. The letters indicate the cloaca (‘C’) and cloacal aperture (‘CA’). (**b**) Eleven-months post-surgery. Animal appearing healthy. (**c**) Eleven-months post-surgery. Cloaca displaying no overt recurrence with a small scar and normal urination.

At 6-weeks post-surgery, the wound had almost healed, with a slight dehiscence of sutures at one position, followed by scab formation (Figure 5a). In spite of the dirty surgical margins, at eleven-months post-surgery, there is no overt regrowth of the tumour and the animal is doing well (Figure 5b,c).

## 3. Discussion

Basal cell tumours (BCTs) are epithelial neoplasms showing no epidermal or adnexal differentiation, with the neoplastic cells morphologically resembling the normal basal cells of the epidermis. BCTs are one of the most common skin neoplasms in dogs and cats, and are almost always benign; with <10% being malignant, specifically basal cell carcinoma (BCC) or basosquamous carcinoma (BSC). BCC is not commonly seen in dogs, is relatively common in cats [1] and is the most common skin malignancy in humans [9]. In cats, the head and neck area are most commonly affected [1], and in humans, the most common site for nodular BCC is the face, whereas superficial BCC has a predilection for the shoulders, chest and back [9]. In contrast, the kangaroo in this case report developed BCC on the cloacal aperture; the anogenital region is not a typical site of BCC development in other species.

Although BCC is typically distinguishable by its characteristic histopathological features, differential diagnoses include squamous cell carcinoma, basosquamous carcinoma and adnexal tumours of follicular differentiation, such as trichoblastoma or trichilemmoma. In humans, immunohistochemistry (IHC) using BerEP4 (which detects EpCAM) is reported to be an effective marker of BCC [10] and IHC panels including BerEP4 and CD10 [11], BerBP4 and EMA [12] or BerBP4 and CD34 [13] can reliably be used as a confirmatory diagnosis of BCC from the afore-mentioned differential diagnoses when needed in cases where the histopathological features are not typical. In the veterinary setting, IHC is not typically used to confirm a diagnosis of BCC, however, CD34 staining has been shown to be useful in distinguishing clear-cell BCC from tricholemomma in cats [14].

In this case report, the BCC presented with an area of central necrosis, which is also seen in some large-sized nodular BCCs in humans [15,16]. In both humans and domestic animals, BCC is considered a low-grade malignancy, characterised by slow growth and low angiogenic potential. However, it is locally invasive, and can show extensive infiltration of the dermis and subcutaneous tissue (in more aggressive cases [17]), as well as epidermal ulceration [1,9]. In addition, although rare, there have been a few cases of metastatic BCC in both humans [18] and cats [19,20].

The gold standard for treatment in both domestic animals and humans is surgical excision, which is typically curative [21,22]. However, recurrence is a risk, with studies reporting recurrence rates of 2–5% in humans [23,24] and as such, long-term follow up of patients is recommended. Recurrence of excised BCCs has also been reported in cats [20]. In human BCC, recurrence is typically associated with incomplete surgical margins, with multifocality, Clark level and depth of invasion found as independent risk factors for positive margins [24,25]. The facial region is the most frequent site of positive margins in humans (9–37%) [25], typically due to surgery being a balance between obtaining safe excision margins and a satisfactory cosmetic result [26]. In this case report, follow up monitoring of the kangaroo has been taking place, and there are no overt signs of recurrence at 11-months post-surgery.

When surgery cannot be performed, electrochemotherapy (ECT) may be considered. In humans, a review of BCCs treated with bleomycin–ECT found a 92% complete response after 2 months, which improved to 100% after re-treatment, with a low risk of recurrence [27]. Successful management with bleomycin–ECT has also been reported in felines with BCC of the nasal planum [28]. Other treatment options used for human BCC patients include radiotherapy (which can also be used in the adjuvant setting), hedgehog pathway inhibitors (HHIs; >90% of sporadic BCCs have mutations in *PTCH1* or *SMO*, allowing intrinsic activation of sonic hedgehog pathway), and immune checkpoint inhibitors such as the monoclonal PD-1 antibody, Cemiplimab-rwlc (FDA-approved for locally advanced BCC patients either previously treated with HHIs or for whom HHI use is not suitable) [29].

The major aetiological factor for the development of BCC development in humans is exposure to UV light, with exposure duration and intensity, cumulative UV dose and skin type all playing a role [9]. However, as 20% of BCCs arise on non–sun-exposed skin, other risk factors include ionising radiation exposure, arsenic exposure, immunosuppression and genetic predisposition [9]. In contrast, the aetiology of BCC in animals is not currently known, although there has been a report of BCC in a dog developing as a complication of chronic solar dermatitis [30]. In cats, there has been an association with the presence of viruses, with detection of two novel papillomavirus types in feline BCC, and a proportion of the neoplastic cells displaying prominent papillomaviral-induced cell changes [20,31]. However, more research is needed to determine whether the virus was causative. In cats, long-haired breeds have been found to be at higher risk of developing BCTs [32], and there are reports of breeds having a predilection for BCC development, including Ragdolls [1], consistent with some human BCC showing genetic predisposition.

## 4. Conclusions

This case report adds to our limited knowledge of tumours in kangaroos, describing the first report of a BCC in a Red kangaroo. It is hoped that further investigations of captive wild animals in zoological facilities or wildlife reserves will enhance our knowledge and understanding of the tumour types developed by these species, thus paving the way for earlier detection and successful management.

## Data Availability

The original contributions presented in this study are included in the article. Further enquiries can be directed to the corresponding author.

## Abbreviations

The following abbreviations are used in this manuscript:

BCC	Basal cell carcinoma
BCT	Basal cell tumour
CD10	Cluster of differentiation 10
CD34	Cluster of differentiation 34
ECT	Electrochemotherapy
EMA	Epithelial membrane antigen
FDA	Food and Drug Administration (USA)
HE	Haematoxylin and eosin
HHI	Hedgehog pathway inhibitor
